# Genome-Wide Identification of the Sulfate Transporter Gene Family Reveals That *BolSULTR2;1* Regulates Plant Resistance to *Alternaria brassicicola* Through the Modulation of Glutathione Biosynthesis in Broccoli

**DOI:** 10.3390/antiox14040496

**Published:** 2025-04-20

**Authors:** Guize Wu, Yunhua Ding, Ning Li, Hongji Zhang, Ning Liu

**Affiliations:** 1 Key Laboratory of Vegetable Biology of Yunnan Province, Yunnan Agricultural University, Kunming 650201, China; 2 Beijing Vegetable Research Center, National Engineering Research Center for Vegetables, Beijing Academy of Agriculture and Forestry Sciences, Beijing 100097, China; 3 State Key Laboratory of Vegetable Biobreeding, Beijing Academy of Agriculture and Forestry Sciences, Beijing 100097, China; 4 Key Laboratory of Urban Agriculture (North China), Ministry of Agriculture and Rural Affairs, Beijing 100097, China

**Keywords:** sulfate transporters, evolutionary analysis, bioinformatics, *Alternaria brassicicola*, *BolSULTR2;1*, glutathione

## Abstract

Sulfate transporters (SULTRs) are key players that regulate sulfur acquisition and distribution within plants, thereby influencing cellular redox hemostasis under pathogen attacks, such as *Alternaria brassicicola* (*Ab*). In this study, a total of 23 *BolSULTR* (*Brassica oleracea* SULTR) genes were identified from the *Brassica* genome. These *BolSULTR*s are distributed across nine chromosomes, with all collinear *BolSULTR* gene pairs undergoing purifying selections. Phylogenetic analysis reveals that the SULTR family is evolutionarily conserved among plant kingdoms. qRT-PCR analysis demonstrated that the expression of *BolSULTR*s varies across different plant organs and is modulated by hormonal signals. Furthermore, transcriptome analysis identified several *BolSULTR*s whose expression levels were depressed in *Ab*-challenged leaves in broccoli. Among them, the *BolSULTR2;1* gene emerged as a key player in the plant’s response to *Ab*. Virus-induced gene silencing (VIGS) of *BolSULTR2;1*s resulted in elevated glutathione (GSH) levels and enhanced tolerance to *Ab.* Taken together, these findings underscore the role of *BolSULTR2;1* in maintaining redox homeostasis and enhancing plant disease resistance, suggesting its potential as a target for genome editing to develop broccoli varieties with improved pathogen tolerance.

## 1. Introduction

Sulfur is one of the essential elements in all living organisms, and it plays a fundamental role in various biochemical processes in higher plants [1]. The leaves and roots of plants are essential tissues for the acquisition of external sulfur (S), playing a crucial role in the plant’s overall nutrient uptake and metabolism. Plants actively absorb hydrogen sulfide (H_2_S) through their leaves as a source of sulfur. The sulfur-deprived *Brassica* plants can be more easily restored to the level of plants grown in the presence of sulfate with H_2_S [2]. Plant roots take up sulfur from the soil mainly in the form of sulfates (SO_4_^2−^), which is commonly mediated by several integral membrane proteins, namely sulfate transporters (SULTRs) [3,4]. Once imported into root cells, sulfate is translocated to plastids in leaves and also via SULTRs, where sulfate is converted to adenosine 5′-phosphosulfate (APS) [5]. Subsequently, APS undergoes two consecutive enzymatic reactions and is sequentially reduced to sulfite and sulfide by APS reductase (APR) and sulfite reductase (SiR), respectively. Finally, sulfide is incorporated into the carbon skeleton of O-acetylserine (OAS), and the resulting product cysteine is the first reduced sulfur donor involved in the biosynthesis of other sulfur biomolecules, such as methionine, sulfolipides, vitamins, glutathione (GSH), and glucosinolates [6,7,8,9].

It is reported that the typical SULTR proteins are generally H^+^/SO_4_^2−^ co-transporters with 12 transmembrane domains (TMDs), a Sulfate_transp domain in the N-terminal region, and an anti-sigma antagonist (STAS) domain in the C-terminal region [10,11]. Thus, the *SULTR* genes can be identified from the plant genome according to the sequences of conserved domains. For example, the model plant *Arabidopsis thaliana* contains 12 *SULTR* genes in its genomes [12,13]. Based on sequence similarity, the SULTR gene family can be divided into four groups. In *Arabidopsis*, Group I includes *SULTR1;1*, *SULTR1;2*, and *SULTR1;3,* known as high-affinity transporters distributed in the root surface cell layer of the roots [14,15]. However, Group II includes *SULTR2;1* and *SULTR2;2*, which are low-affinity transporters responsible for sulfate transport from root to stem. It has been accepted that SULTRs in Group II mainly participate in long-distance transport, maintaining sulfur metabolite levels between the aerial parts [16]. Group III is the largest group with preferential expression in leaves, but their exact roles remain under investigation. It is proposed that SULTR3s facilitate SO_4_^2−^ uptake into chloroplasts and consequently influence downstream sulfate assimilation [17,18]. Interestingly, a yeast-based complementation experiment suggested that SULTR3;5 could directly interact with SULTR2;1, enhancing the transporter ability of SULTR2;1 under sulfur deficiency [19]. *SULTR4;1* and *SULTR4;2* belong to Group IV, and these SULTR4s are localized to the vacuolar membrane, where they actively export SO_4_^2−^ from the vacuole to the cytoplasm [20]. Thus, these functionally diversified SULTRs play central roles in the absorption and distribution of sulfate within different plant organs, cell types, and subcellular compartments.

Intensive studies have demonstrated the critical roles of *SULTR* genes in plant growth and development. In *Arabidopsis*, sulfur starvation strongly activates the expression of *SULTR1;1*, whereas under normal sulfate supply, SULTR1;2 is the major transporter [14,21]. Sulfate translocation from roots to shoots is largely mediated by *microRNA395* (*miR395*) [22], which affects sulfate transport from root to stem and from old leaves to new leaves via direct suppression of *SULTR2;1*. The *Arabidopsis sultr2;1* mutant exhibits an early flowering phenotype while keeping plant biomass unchanged [23], whereas several *sultr3* mutants display stunted growth to varying degrees, resulting in a smaller thallus and shorter roots due to the reduced sulfate uptake to chloroplasts [17,24]. The SULTR4;1 and SULTR4;2 transporters are associated with sulfate transport in the vacuole, fulfilling the S requirements in young leaves and seeds [25,26]. Vacuoles isolated from callus of the *sultr4;1* and *sultr4;2* double knockout showed excess accumulation of sulfate [27]. However, our knowledge about their functions under abiotic and biotic stresses is still scarce. In rice, the *miR395*-*OsSULTR2s* module also regulates sulfate accumulation in leaves, resulting in broad-spectrum resistance to bacterial pathogens [28]. In the model legume *Medicago truncatula*, the majority of *MtSULTR3* genes were strongly upregulated by drought or salinity [29], implying that they might be a potential target for abiotic stresses. Indeed, the loss of *SULTR3;1* function led to lower ABA levels in *Arabidopsis* seedlings [30], further indicating that SULTRs could be the cross-talking point between sulfur metabolism and stress signaling pathways. Those findings suggest that SULTRs could help plants maintain sufficient sulfur supply, thereby protecting plants from environmental stresses as well as pathogen attacks.

Broccoli (*Brassica oleracea* L. var. *italica*) is a cruciferous vegetable that is widely cultivated worldwide owing to its nutritional value [31,32], and, in 2023, global production of broccoli (cauliflower included) reached approximately 25.9 million tons (http://faostat.fao.org/ (accessed on 15 October 2024)). Black spot disease, a necrotrophic pathogen caused by fungal *Alternaria brassicicola* (*Ab*), severely affects broccoli production. So far, fully *Ab*-resistant broccoli cultivars have not been reported yet, and, currently, all commercial varieties are highly susceptible to *Ab* [33]. Therefore, genetic manipulation is the most efficient method for the rapid improvement of broccoli resistance to *Ab* [34]. As sulfur is implicated in plant immunity, modulation of sulfur transport might benefit plant sulfur metabolism and consequently confer *Ab* resistance to broccoli.

Although the *SULTR* genes have been investigated in several plant species, their genomic distribution and biological functions in broccoli, to our knowledge, have been largely unexplored. Therefore, in this study, a genome-wide, comprehensive analysis of *SULTR* genes was performed to search for SULTRs associated with broccoli *Ab* resistance. In total, 23 *SULTR* genes were identified from broccoli and further confirmed through sequencing. The physical and chemical characteristics, genomic structures, chromosomal locations, evolutionary relationship, and expression profiles of the broccoli *SULTR* gene family were examined in detail. Furthermore, the roles of *BolSULTR2;1a* were analyzed through the virus-induced gene silencing (VIGS) approach, supporting its critical role in *Ab* resistance. Our study provides molecular information regarding the *SULTR* gene family in broccoli, providing a theoretical foundation for future genetic engineering to generate *Ab*-resistant vegetables.

## 2. Materials and Methods

### 2.1. Identification and Characterization of BolSULTRs

The newest *Brassica oleracea* cv. JZS V2.0 data were obtained from the Brassicaceae Database (BRAD) (http://brassicadb.cn (accessed on 10 December 2024)) [35]. The genome CDS sequence data were extracted and transformed into protein sequences using TBtools-II v2.154. Then, all *Arabidopsis thaliana* SULTR protein sequences were downloaded from the Arabidopsis Genome Database (TAIR) using SULTR as a keyword. These sequences were then used as seed sequences to search the *B. oleracea* protein database for the candidate BolSULTR using BLASTp with an e-value ≤ 1 × 10^−5^ [36]. In addition, the *B. oleracea* protein sequences of *SULTR* were blasted by using Hidden Markov Models (HMMs) for Sulfate_transp (PF00916) and STAS (PF01740) domains downloaded from the Pfam database [37]. Domain structures were verified using NCBI-CDD and the Pfams database [38,39], yielding 23 SULTR genes. Physicochemical properties were predicted using ExPASy (http://web.expasy.org/protparam (accessed on 10 November 2024)) [40], and subcellular localization was predicted using Plant-mPLoc [41].

### 2.2. Phylogenetic Analysis, Classification, and Evolutionary Analysis

Protein sequences of *SULTR* genes were retrieved from TAIR and BRAD. Multiple sequence alignment of broccoli and *Arabidopsis SULTR* proteins was performed using MUSCLE in MEGA X. Phylogenetic trees were constructed through the neighbor-joining method with 1000 bootstrap replicates [42]. Based on their kinship with *Arabidopsis*, the broccoli *SULTR* genes were designated as *BolSULTR*. Tree visualization and subfamily classification were performed using iTOL (https://itol.embl.de/login.cgi (accessed on 11 December 2024)). The neighbor-joining tree was constructed based on the alignment of SULTR amino acid sequences from *Chlamydomonas reinhardtii*, *Physcomitrella patens*, *Oryza sativa*, *Solaunum tuberosum*, *Solanum lycoperiscum*, *Brassica oleracea* L. var. *italica*, and *Arabidopsis thaliana*. The percent bootstrap support for 1000 replicates is shown on each branch.

### 2.3. Chromosome Location and Homologous Gene Analysis

The chromosome location of *BolSULTR* genes appeared on the BRAD. The density of each gene was analyzed in the entire chromosome region. We divided the number of genes by the region size and then multiplied it by a conversion factor of 1,000,000 to obtain the number of genes per million base pairs (Mb). Moreover, gene duplication events were examined using MCScanX with default parameters [43]. The events of gene duplication in broccoli were revealed through the Advanced Circus tool from TBtools. Meanwhile, the synonymous mutation rate (Ks), the nonsynonymous mutation rate (Ka), and the ratio of nonsynonymous to synonymous mutation rates (Ka/Ks) for duplicated *BolSULTR* genes were calculated using the Ka/Ks Calculator tool in TBtools. In addition, we downloaded the genome annotation files for *Arabidopsis thaliana* and rice from the Ensembl Plants database (https://plants.ensembl.org/index.html (accessed on 10 November 2024)) to create synteny maps and analyzed the distribution of homologous genes across these different species by using the TBtools.

### 2.4. Analysis of Conserved Motifs and Gene Structures of the BolSULTRs

We conducted a protein motif analysis of *BolSULTR* family members utilizing the MEME tool (http://meme-suite.org/tools/meme (accessed on 11 December 2024)) with default settings. Briefly, motif sites were expected to be distributed in sequences with zero or one occurrence per sequence, and a maximum of 12 patterns were expected. Other settings were as follows: the motif E-value threshold: no limit; sites per motif: 16–23; and motif width: 15–50. The motifs were annotated in the form of amino acid logo plots. The quantitative structures of exons and introns were determined using the genome annotation file (GFF file format). TBtools was utilized to integrate and visualize phylogenetic relationships, motif patterns, and gene structures.

### 2.5. Promoter Cis-Acting Element

We used TBtools to extract promoter sequences of upstream 2000 bp *BolSULTR* genes, and the cis-regulatory elements were identified using PlantCAR [44]. The redundant elements were removed, and, according to its potential functions, all elements were classified. TBtools was used to visualize element distribution (Figure S3).

### 2.6. Protein–Protein Interaction Prediction

The BolSULTR protein interactions based on data from *Arabidopsis* orthologs were analyzed using the online tool STRING (https://string-db.org/ (accessed on 11 November 2024)). The interacting proteins were functionally characterized through Gene Ontology (GO) and Kyoto Encyclopedia of Genes and Genomes (KEGG) pathway enrichment analyses.

### 2.7. Plant Material and Treatment

Seeds of the susceptible cultivar ‘41-3’ of *Brassica oleracea* L. var. *italica* (broccoli) were germinated in a soil–perlite mixture (2:1) in a plant growth chamber with a photoperiod of 16 h of light and 8 h of darkness at a temperature of 25 °C ± 1 °C and a relative humidity of approximately 65%. The 8-week-old broccoli seedlings were used for Ab infection experiments. The *A. brassicicola* wild-type strain used in this study was routinely grown and maintained on potato dextrose agar at 24 °C.

### 2.8. Expression Patterns of BolSULTR Genes Based on RNA-Seq Data

The concentration of the *A. brassicicola* conidial suspension was 5 × 10^5^ spores per milliliter with 0.05% (*v*/*v*) Tween 20 was added as a surfactant, which was applied using a spray bottle directly onto each leaf. The control group received the same volume of Tween 20 as the treatment groups. Additionally, the relative humidity of the environment was increased to 80%. We selected leaves at different time points (0 days, 1 day, 3 days, 5 days, and 7 days) after treatment to perform transcriptome sequencing (RNA-Seq); each time point included three samples, with each sample consisting of four biological replicates. Additionally, we searched seven RNA-seq datasets of *Brassica* from the GSE42891 (GEO database), specifically for the root, stem, leaf, flower, silique, bud, and callus. The *BolSULTR* genes were used as a template to screen for highly homologous genes in RNA-seq datasets, and then the expression pattern of the target gene was extracted based on the FPKM value. Subsequently, log2 transformation was applied to generate a heatmap using TBtool. RNA-seq dataset information is presented in Supplementary Tables S4 and S5.

### 2.9. Expression of SULTR Genes in Broccoli Treated with Different Phytohormones

We used different phytohormones to treat broccoli seedling leaves. The exogenous hormones were dissolved in a small amount of ethanol and then diluted to the desired concentration for each treatment with distilled water, including 100 mg/L of Ethephon (ETH, Solarbio, 16672-87-0, Beijing, China), 1 mM/L of MeJA (Macklin, 39924-52-2, Beijing, China), and 1 mM/L of SA (Biotopped, 69-72-7, Beijing, China). Working fluids were applied to the plants through spraying and distilled water was used as a control treatment while ensuring that the concentration of anhydrous ethanol in the spray solution was the same. Additionally, 0.1% (*v*/*v*) Tween 20 was added as a surfactant. We ensured that each plant leaf was sprayed thoroughly until it dripped completely. For each treatment, we selected three samples at different time points (0 h, 4 h, 24 h), and for each sample we used 3 biological replicates. The samples were verified through qRT-PCR. These samples were then rapidly frozen in liquid nitrogen and stored at −80 °C for further experiments.

### 2.10. RNA Extraction and qRT-PCR Analysis

Total RNA was extracted from young broccoli leaves using the FastPure Plant Total RNA Isolation Kit (Vazyme, Nanjing, China), with modifications [45]. RNA was then treated with DNase I to remove genomic DNA in one step, and 3 μg of total RNA was used for first-strand cDNA synthesis using a cDNA synthesis kit (Transgen, Beijing, China). The resulting cDNA sequences served as templates for quantitative reverse transcription real-time PCR (qRT-PCR) using a Roche LightCycler 480 II and the LightCycler 480 SYBR Green I Master Mix (Toyobo, Shanghai, China), following the manufacturer’s instructions. Fold changes were calculated from 2^−ΔΔCt^ values, with each assay replicated at least three times using three biological replicates (independent RNA preparations). The *Actin-2* was employed as a reference gene. The relevant primer sequences are presented in Supplemental Table S3.

### 2.11. Virus-Induced Gene Silencing (VIGS) of BolSULTR2;1

We used the tobacco rattle virus (TRV) vector system, consisting of pTRV1 and pTRV2 vectors, to achieve virus-induced *BolSULTR2;1* silencing (VIGS). The 315 bp sequence from CDS was subcloned into the viral vector pTRV2. The *Agrobacterium tumefaciens* strain GV3101 harboring the recombinant plasmids pTRV2:*BolSULTR2;1* and pTRV1 was used for in vitro agroinoculation through leaf injection. The *Brassica oleracea* 15-cis-phytoene desaturase (PDS) was used as a positive control, and the negative control was the pTRV2 empty vector (TRV:00). The *Agrobacterium tumefaciens* strain GV3101 carrying the constructs was cultured overnight at 28 °C and centrifuged to remove culture medium containing antibiotics when OD_600_ reached 1.5–2.0. The concentrated bacterial solutions were fully suspended with 1 mL of 10 mM magnesium chloride solution containing 200 μM of acetylsyringone and then diluted into a working solution (OD_600_ = 0.5). Then, a needleless syringe was used to infiltrate the abaxial surface of the broccoli cotyledons. Each treatment was inoculated with fifty broccoli seedlings. All of the plants were grown in the same growth environment at 25/23 °C (day/night), with a 16 h light/8 h dark cycle.

When observing a bleached leaf phenotype in seedlings infiltrated with *pTRV:PDS*, we performed a qRT-PCR analysis on the selected seedlings (pTRV:*BolSULTR2;1*). Significant downregulation of *BolSULTR2;1* in the bleached seedlings compared to negative controls indicated successful silencing. At that moment, we collected a sufficient amount of leaf tissue for further analysis. Subsequently, the silenced seedlings and control plants with comparable growth conditions were selected for inoculation with the *Ab* suspension liquid (5 × 10^5^ conidia/mL). Determination of the stress-related physiological index contents of the leaves was performed on the fifth day after inoculation, and ImageJ software (Ver. 1.54k) was used to calculate the area of the lesions. The VIGS experiments were repeated at least three times, and each treatment was applied to more than 15 plants. The primers used for vector construction are listed in Supplemental Table S3.

### 2.12. Measurements of ROS-Related Physiological and Biochemical Parameters

Quantifications of reduced glutathione (GSH), superoxide dismutase (SOD), catalase (CAT), and ascorbate peroxidase (APX) were conducted using commercial kits (BC1175 for GSH, BC0175 for SOD, BC0205 for CAT, and BC0225 for APX) purchased from Solarbio Life Sciences Co., Ltd. (Beijing, China) following the manufacturer’s instructions. The spectrophotometry was performed with an Agilent BioTek microplate reader (Santa Clara, CA, USA). Each assay was performed with three biological replicates. The detection of reactive oxygen species (ROS) was conducted using Coolaber DAB (3,3′-diaminobenzidine) detection kit (Beijing, China). The leaf staining procedure was conducted as previously described [46].

## 3. Results

### 3.1. Identification of SULTR Genes in Brassica Oleracea Broccoli

In this study, 23 unique *BolSULTR* members were obtained from broccoli and named according to their homology with *Arabidopsis* counterparts (Figure S1 and Table S1). Information on the gene name, loci number chromosomal location, and physicochemical characteristics of putative proteins of individual *BolSULTR* genes is listed in Table 1. Of these, the molecular weight of putative BolSULTR proteins ranged from 43.33 kDa (BolSULTR3;1c) to 108.00 kDa (BolSULTR4;2). Furthermore, the range of isoelectric points extends from a slightly acidic pI of 5.96 (BolSULTR3;1b) to a more basic pI of 9.63 (BolSULTR3;4b). Notably, except for BolSULTR3;1b protein, which was distinguished by its hydrophilic character, the other members, which exhibit positive GRAVY values, showed a generally hydrophobic nature. Consistent with transporter roles, 19 BolSULTR proteins were predicted to be localized on the cell membrane, while all BolSULTR4 members might be localized to the chloroplasts or vacuoles. Surprisingly, BolSULTR3;1b could be targeted to the nucleus, probably reflecting its distinct functions other than as a transporter. Together, the results reinforced the assumption that these BolSULTRs might functionally diverge and participate in different biochemical processes under disparate environments.

### 3.2. Phylogenetic Analysis of SULTR Gene Family

**Figure 1:**
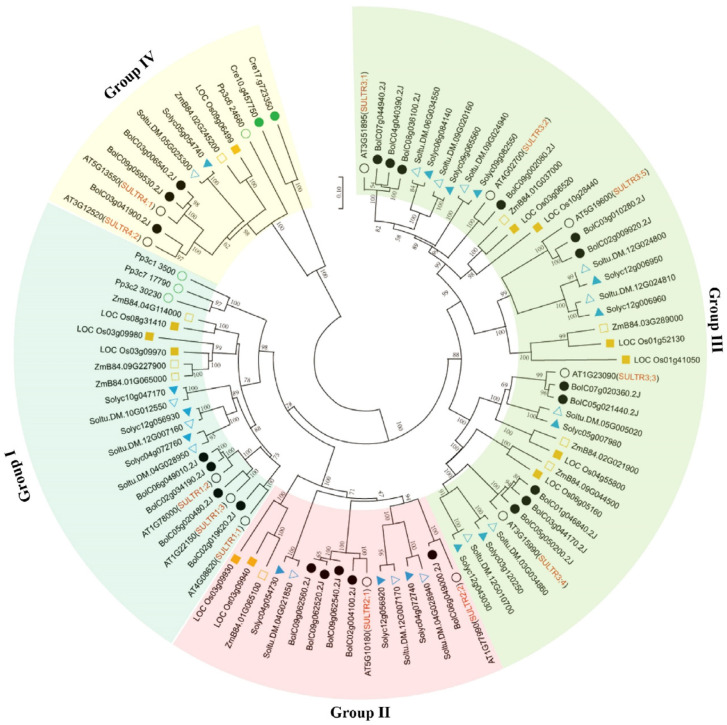
Phylogenetic analysis of SULTR proteins in algae, liverwort, moss, and selected flowering plants. The neighbor-joining tree was constructed based on the alignment of SULTR amino acid sequences from *Chlamydomonas reinhardtii* (green circle), *Physcomitrella patens* (empty green circle), *Solanum tuberosum* (blue triangle), *Solanum lycopersicum* (empty blue triangle), *Oryza sativa* (empty yellow square), *Zea mays* (yellow square), *Brassica oleracea* L. var. *Italica* (black circle), and *Arabidopsis thaliana* (empty black circle). The position of the root was determined from an outgroup consisting of two SULTR-like sequences from yeast (*Saccharomyces cerevisiae*). The percent bootstrap support for 1000 replicates is shown on each branch.

To investigate the phylogenetic relationship between the members of the BolSULTR family, a neighbor-joining tree was constructed based on the multiple alignment of SULTR protein sequences from green algae (*Chlamydomonas reinhardtii*), bryophyte (*Physcomitrium patens*), green algae (*Chlamydomonas reinhardtii*), rice (*Oryza Stativa*), tomato (*Solanum tuberosum*), potato (*Solanum lycopersicum*), *Arabidopsis* (*Arabidopsis thaliana*), and broccoli. Among these subfamilies, subclade III stands out with 34 members, which makes it the largest group; subclade I includes 26 members, followed by subclade IV (16 SULTRs) and subclade II (11 SULTRs). Only two and four SULTR proteins were identified from the genome of green algae and bryophytes, respectively (Figure 1). The reduced number of SULTRs in lower photosynthetic organisms may reflect evolutionary adaptations to their specific environmental conditions and physiological requirements. Conversely, in higher plants, such as *Arabidopsis*, tomato, and rice, dozens of SULTRs were found in their genomes, which indicates the expansion of the SULTR gene family in higher plants.

### 3.3. Conserved Domains of BolSULTRs

To better understand the structural similarity of these SULTRs, the Hidden Markov Models were used to identify the TMD, STAS, and other conserved domains of BolSULTR, and the predicted domains were visualized in ChiPlot (Figure S2). Almost all BolSULTR proteins possess the Sulfate_transp domain (PF00916) and the STAS domain (PF01740), indicating that the BolSULTRs are highly conserved in broccoli. However, SULTR2;2 only has the intact TMD region, and its STAS domain was abolished. Thus, it is plausible that the transporter ability of SULTR2;2 could be impaired due to the sequence truncations. Other domains were identified in some BolSULTRs, which might confer novel functions. For example, in addition to TMD and STAS, BolSULTR4;2 also contains the Chitin-binding and glycoside hydrolase family 19 domains in its C-terminal. Similarly, LSm7 RNA-binding and high mobility group (HMG)-box motifs were found in BolSULTR3;1a and BolSULTR3;1b, respectively (Table S1).

### 3.4. Analysis of Conserved Motifs and Gene Structure of BolSULTRs

**Figure 2:**
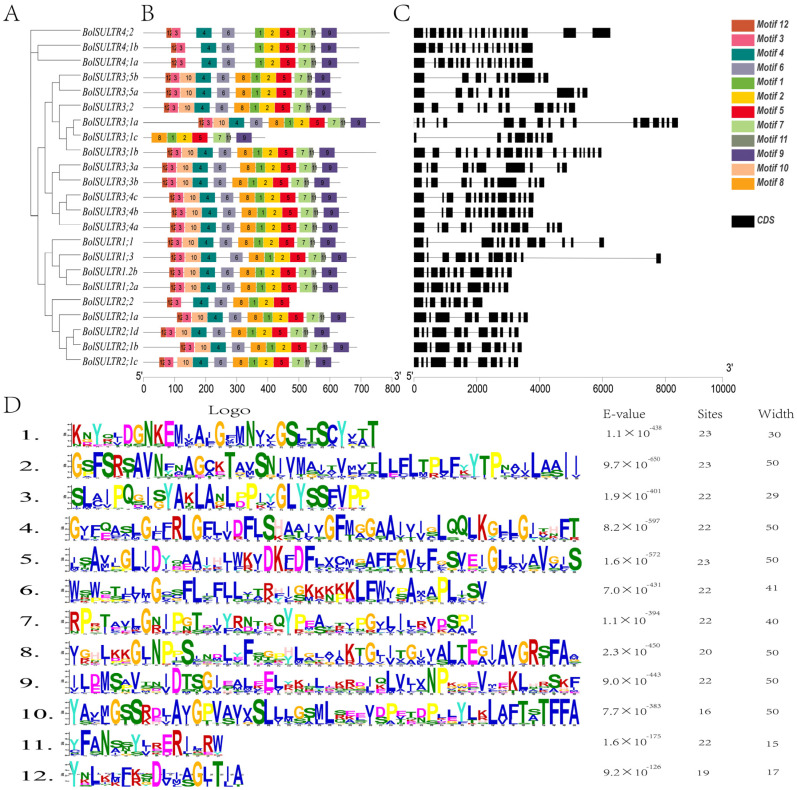
Conserved motifs and gene structural analyses of the *BolSULTRs*. (**A**) Creating a phylogenetic tree involved arranging 23 BolSULTR conserved motifs. (**B**) The two conserved domains are divided into 12 motifs. The specific motifs or sequence patterns are retained across different *BolSULTR* proteins due to their functional importance. (**C**) General gene structure of BolSULTRs. Black lines and black boxes indicate introns, exons, and UTR, respectively. (**D**) The functional active site or binding site corresponds to the motif’s detailed sequence.

A total of 12 different motifs were detected among the BolSULTR proteins, with lengths ranging from 15 to 50 amino acids. The number of motifs varied from 7 to 12 among 23 BolSULTR proteins. The motifs 1, 2, and 5, containing the Sulfate_transp motif, or motifs 7, 9, and 11, containing the STAS signature motif, were characteristic in all BolSULTR proteins. These findings are consistent with the domain analysis of the SULTR family (Figure 2B,D). Additionally, motif 8 was exclusively absent in Group IV, and several motifs were deleted in BolSULTR2;2, BolSULTR3;1c, implying the different functions of these BolSULTRs. Additionally, most *BolSULTRs* within the same subfamily generally exhibit similar genetic structures in terms of the number and length of introns and exons (Figure 2C). *BolSULTR2;1* members might arise from recent duplication events, as evidenced by the extremely high similarity in gene structure and sequences. In Group III, the number of exons ranged from 8 to 21, exhibiting a complex pattern. The *SULTR* members in Group IV had 15 to 19 exons, and *BolSULTR4;2* possessed 2 longer exons at the 3′ end due to the additional domains. Overall, the conserved motifs and complex gene structure might provide more chances of heredity and variation for *BolSULTR* genes.

### 3.5. The Chromosome Localization, Gene Duplication, Ka/Ks, and Synteny Analysis

**Figure 3:**
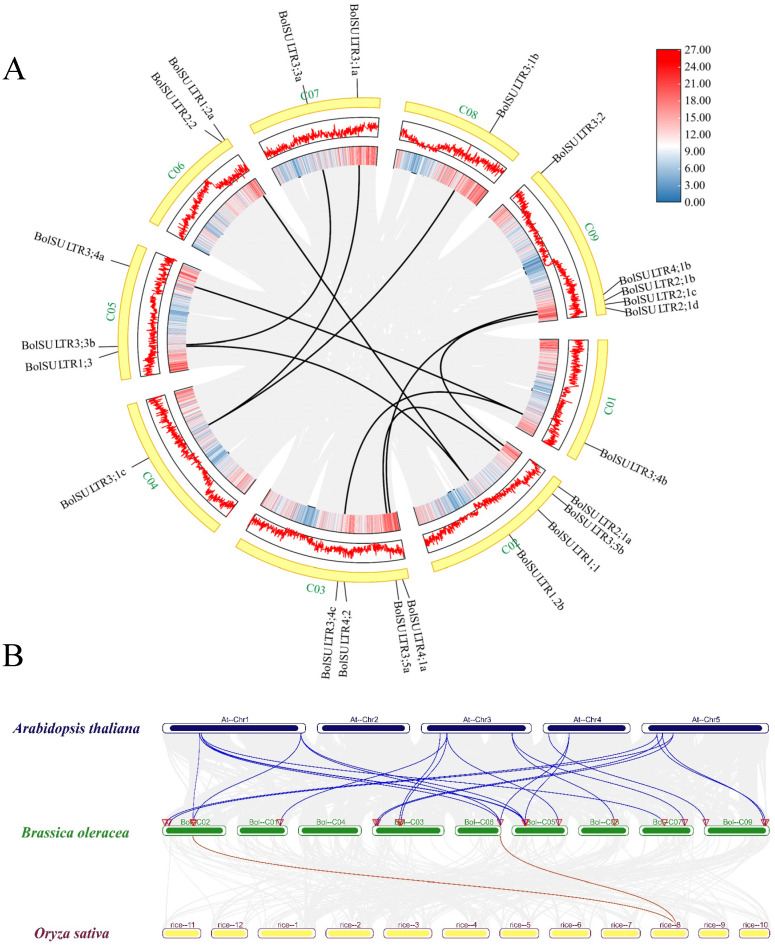
Synteny analysis of *BolSULTRs* in the Brassica oleracea genome and between different species. (**A**) Chromosomal locations and synteny analysis of *BolSULTRs* in broccoli. Gray lines represent all synteny blocks in the *B. oleracea* genome, while black lines indicate segmental duplication genes between *BolSULTRs*. Chromosome gene density is represented by a hot map (inner circle) and column map (middle circle), with the outer circle showing the length of chromosomes. (**B**) Cognate relationship of *BolSULTRs* in broccoli, *Arabidopsis*, and rice; colored lines emphasize the pairs of syntenic *BolSULTRs*.

The results showed that 23 *BolSULTR* genes were unevenly distributed in nine chromosomes (Figure 3A). The majority of *BolSULTR* genes were mainly located on chromosomes C02, C03, and C09. Notably, only one *BolSULTR3* gene was found on chromosomes C01, C04, and C08, while most *BolSULTR2*s were clustered on chromosome C09. In addition, the collinearity relationships showed *BolSULTR1;1*, *BolSULTR2;2*, *BolSULTR3;2*, and *BolSULTR4;2* without associated collinear genes and a high degree of functional conservation among cruciferous species (Figure 3B). This may be due to biased gene loss in redundant gene members lacking collinear relationships [47]. Furthermore, consistent with previous observations, all collinear *BolSULTR* gene pairs had a Ka/Ks ratio of less than one, indicating that these genes underwent purifying selection. Interestingly, the genes *BolSULTR2;1a* and *BolSULTR2;1b* were involved in segmental duplication, while *BolSULTR2;1b*, *BolSULTR2;1c*, and the *BolSULTR2;1d* resulted from tandem duplication (Figure 3A and Table S2).

### 3.6. Analysis of the Promoter Region of the BolSULTRs

**Figure 4:**
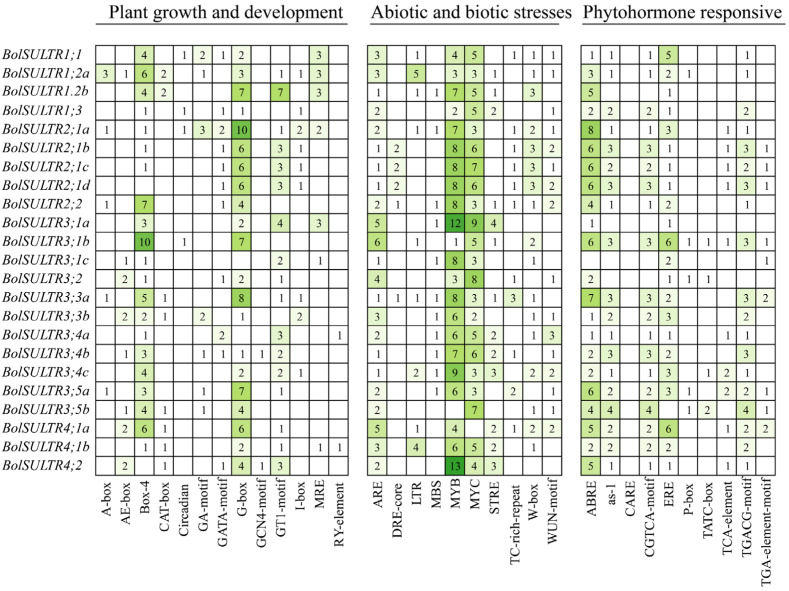
Analysis of cis-acting elements of 23 *BolSULTR* gene promoters. Cis-acting elements of the 23 *BolSULTR* gene promoters are grouped, including phytohormone-responsive, biotic/abiotic stress, and plant growth and development. The different colors and number of boxes indicate different promoter elements in the *BolSULTR* genes.

Through PlantCare analytics, we can identify the potential regulatory regime through the cis elements in the 2 kb upstream of all identified *BolSULTR* genes. It was clear of cis-acting element distribution (Figure S3), and we drew on their functional annotations to categorize three types: plant growth and development, abiotic and biotic stresses, and phytohormone responses (Figure 4). The results of type I showed that *BolSULTR* genes were associated with plant growth and development. This is because there were abundant optical signal sensing and transduction elements distributed in the promoter region, like Box-4 (ATTAAT), G-box (TACGTG), and GT1-motif, which can regulate gene expression related to photomorphogenesis. Important cis-acting elements for the cell cycle and metal ion response were found, such as CAT-box and MRE. These elements were also related to responses to environmental changes.

The abiotic and biotic stress response elements were divided into the second category, which is associated with stress responses, particularly those related to defense against pathogens and other environmental stresses. Among them, the elements responding to abiotic stress were widely distributed, including MYB (CCAAT), MYC (CACATG), ARE (anaerobic response element), MBS (drought inducibility), and LTR (low-temperature-responsive) binding sites. The second was the functional element in response to biological stress, including TC-rich-repeat, W-box (WRKY-box), WUN-motif, and STRE (Stress-Responsive Element). These elements are involved in the activation of *BolSULTR* genes that are part of the plant’s response to various biotic stresses. The phytohormone response elements were grouped into the third category, which mainly included ABRE (ABA-responsive element), CGTCA-motif (MeJA-responsiveness), TGACG-motif (cis-acting regulatory element involved in MeJA-responsiveness), as-1element, and TCA-element (salicylic acid responsiveness). Additionally, ERE (Ethylene Response Element), TGA-element (auxin-responsive element), and P-box (gibberellin-responsive element) were identified in those *BolSULTR* promoters. These motifs are crucial for the regulation of genes involved in secondary metabolite production, defense compound biosynthesis, and other protective responses. Together, cis-element analyses indicate that *BolSULTRs* might be regulated by light, hormones, stress responses, and developmental cues.

### 3.7. BolSULTR Interaction Prediction

**Figure 5:**
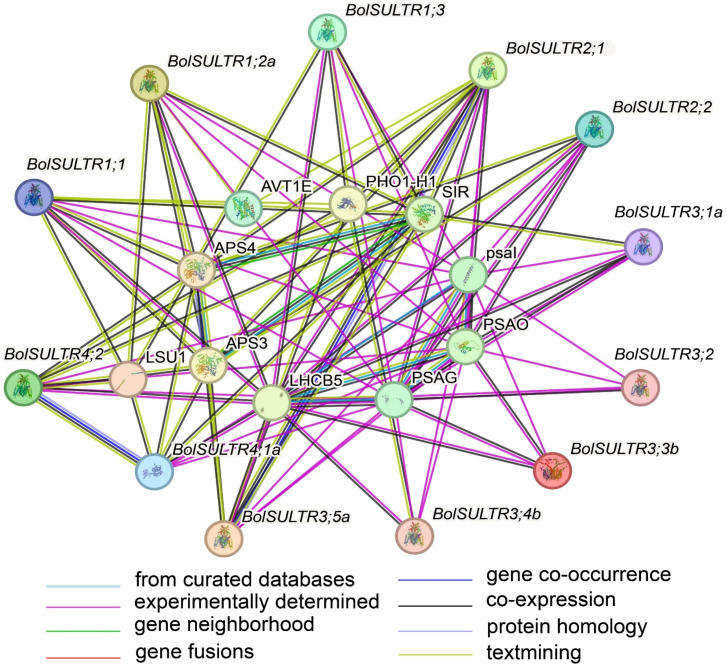
Interactions between BolSULTR proteins and other proteins. Line color indicates the type of interaction evidence; edges represent protein–protein associations.

As interacting objects, amino acid transporter AVT1E (the amino acid/polyamine transporter 2 family), LHCB5 (Chlorophyll a-b binding protein), PSAO (Photosystem I subunit O), and PSAG (Photosystem I reaction center subunit V) were predicted to be the potential partners of BolSULTRs (Figure 5 and Table S6), which suggests that sulfur assimilation is closely associated with the antioxidant defense mechanisms of photosynthesis [48]. As expected, the BolSULTR proteins could interact with some proteins related to sulfur metabolism, including APS4 (ATP sulfurylase 4) and SIR (assimilatory sulfite reductase). This shows that BolSULTR proteins could form complexes with other proteins involved in the sulfur assimilation pathway to enhance efficiency and regulation.

### 3.8. Expression Patterns of the BolSULTR Genes

**Figure 6:**
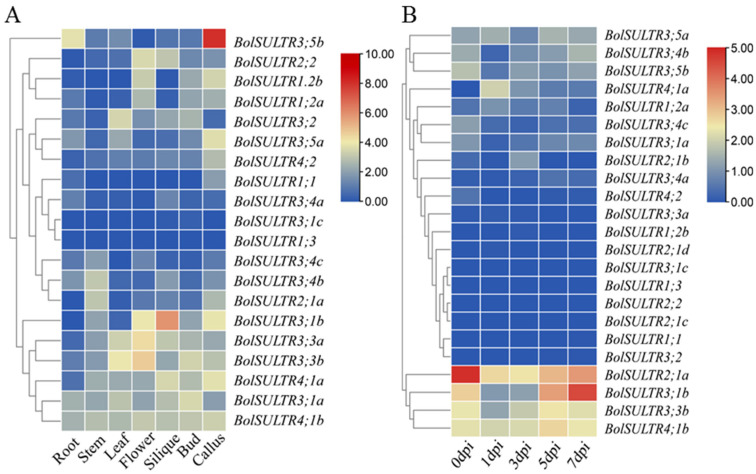
Expression patterns of the *BolSULTR* genes. (**A**) Different tissues’ and organs’ expression of *BolSULTR* genes; RNA-seq datasets belong to the GSE42891 (GEO database). (**B**) Expression of the *BolSULTR* genes under *Ab* challenges at different points in time (0, 1, 3, 5, and 7 days). The transcriptome data were averaged through log2 transformation and TBtools software to generate a heatmap. The FPKM values are from three independent biological replicates.

The expression profiles of *BolSULTR* were obtained from transcriptome analysis. Expression patterns of *BolSULTR* genes in different organs and under the *Ab* are shown in Figure 6A,B. The seven RNA-seq datasets of *Brassica* told us that *BolSULTR3;1a* and *BolSULTR4;1b* were ubiquitously expressed throughout the different organs, whereas other genes exhibited organ-preferential patterns. Specifically, *BolSULTR2;1a* was highly expressed in the stem. *BolSULTR3;1b*, *BolSULTR2;2,* and *BolSULTR3;3b* were highly expressed in flowers and the silique, and *BolSULTR3;5b* was predominantly expressed in the callus and roots. In contrast, *BolSULTR3;1c*, *BolSULTR1;3*, *BolSULTR1;1,* and *BolSULTR3;4a* displayed relatively lower expression levels across all organs. In summary, the expression of *BolSULTR* genes could be detected in diverse tissues and organs. The transcriptome datasets showed that initial gene expression levels (*BolSULTR2;1a*, *BolSULTR3;1a/b*, *and BolSULTR4;1b* and *BolSULTR3;3b*, *BolSULTR3;4b*, and *BolSULTR3;5b*) exhibited much higher expression levels than the other *BolSULTRs*. After the broccoli plants were challenged with *Ab*, their gene expression levels were robust biology stress responses and remained low until a correction occurred later in the course of the disease, particularly for *BolSULTR2;1a*. But, the homologous genes of *BolSULTR2;1b/c/d* did not respond. This suggests that *BolSULTR2;1a* may be important in early infections. Furthermore, the expression of *BolSULT3;1b* reached its maximum at 7 dpi, implying that it might be late-responsive genes during *Ab* infections.

### 3.9. Expression of BolSULTR Genes Under the Phytohormone Treatments

**Figure 7:**
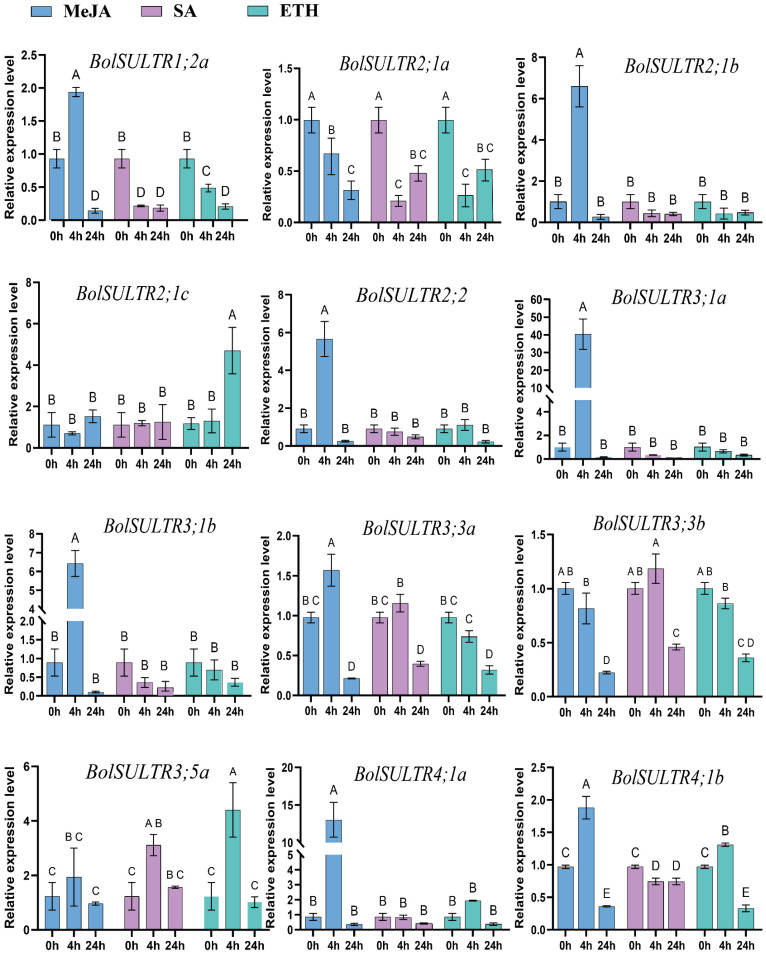
The expression levels of *BolSULTRs* in different hormones using qRT-PCR analysis. In total, 100 mg/L of Ethephon, 1 mM/L of MeJA, and 1 mM/L of SA were sprayed onto seedling leaves. The leaves of mature broccoli were selected at different time points (0 h, 4 h, 24 h) and three from independent biological replicates. Capital letters indicate significant differences (*p* < 0.01). Error bars represent the standard deviation.

To further investigate the potential roles of the *BolSULTR* gene family in response to the effects of different hormones on *BolSULTR* expression in broccoli, we selected and examined the relative expression levels of twelve *BolSULTR* genes after various hormone treatments for 0 h, 4 h, and 12 h using qRT-PCR (Figure 7). The results indicate that the expression of most *BolSULTR* genes was upregulated robustly under exogenous MeJA treatment conditions, suggesting that MeJA could induce plant resistance to saprophytic pathogens [49,50,51]. Interestingly, the *BolSULTR2;1a* gene was downregulated in response to various hormones.

### 3.10. Silencing of BolSULTR2;1 Resulted in Reduced Leaf Spot Stress

**Figure 8:**
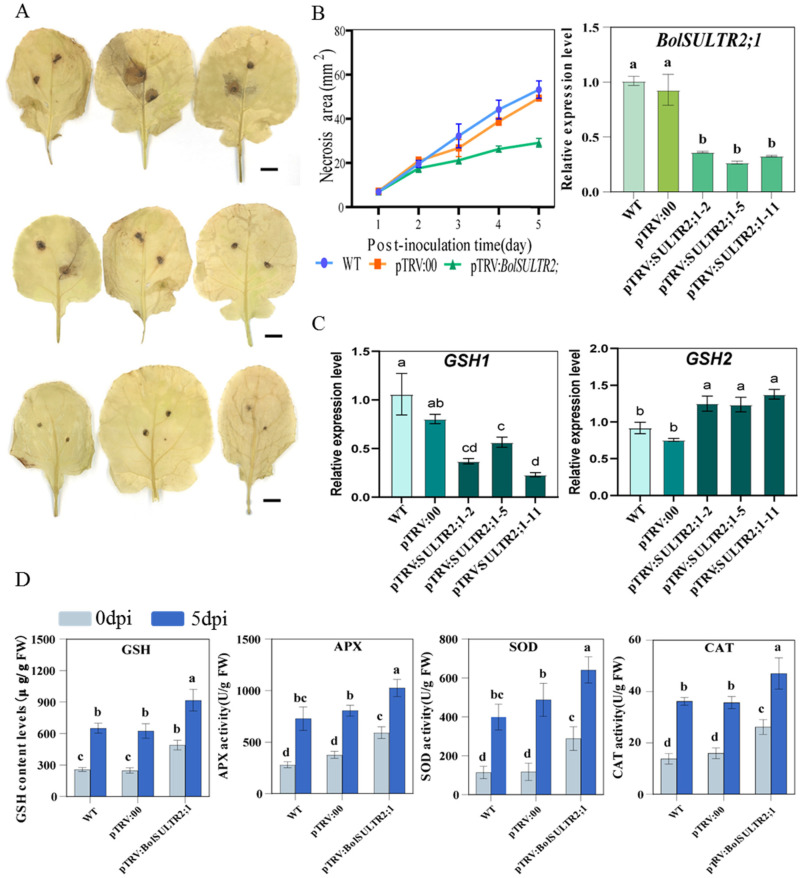
Physiological parameter measurements of *BolSULTR2;1*-silencing and control plants. (**A**) DAB reactive oxygen species staining in leaves. Bars = 2 cm. (**B**) Analysis of infection site area; *BolSULTR2;1* was specifically downregulated in silent plants. (**C**) Analysis of expression level of glutathione synthetases gene. (**D**) Physiological parameters of GSH, APX, SOD, and CAT with comparison between 0 days and 5 days under *Ab* stress in *BolSULTR2;1*-silencing plants. The mean values were derived from three independent biological replicates. Letters above each column indicate significant differences (*p* < 0.05 and *p* < 0.01). Error bars represent the standard deviation.

To further verify the functions of the *BolSULTR2;1a* gene in broccoli immunity, we obtained 25 independent silent plants of pTRV:*BolSULTR2;1* via the VIGS approach. The qRT-PCR analysis suggested that the genes’ expression of *BolSULTR2;1* was specifically downregulated in silent plants compared with the control group, and glutathione synthetases2 (GSH2) gene expression was upregulated (Figure 8C and Figure S5B). Then, these silent plants were subjected to phenotypic analysis and physiological parameter measurements. After 5 days of *A. brassicicola* inoculation, the silent plants exhibited local necrosis, and the areas of the necrotic spots were significantly smaller than those of the control plants. DAB staining revealed less extensive lesions of reactive oxygen species (ROS) in the silent plant leaves (Figure 8A). As glutathione (GSH) is the major antioxidant molecule in plants, we measured the GSH (glutathione) content in the leaves’ tissue surrounding the lesion. Consistent with the observed phenotypes, the GSH levels in silent plants were much higher than those of the control. And, with the development of the disease, the activity of various antioxidant enzymes in the silenced plants increased to a higher level than that of the control group (Figure 8D). Thus, these results illustrated that the repression of *BolSULTR2;1* could lead to enhanced tolerance under *Ab* infections.

## 4. Discussion

### 4.1. The Paradox Between Genome Complexity and SULTR Copy Number in Broccoli

Plants use sulfate (SO_4_^2−^) as a primary sulfur source to produce a wide range of secondary organic metabolites that have imperative roles in plant development, metabolism, and stress responses [52]. Once sulfate is acquired from the rhizosphere, it is assimilated to biosynthesize cysteine and other sulfur-containing metabolites in the leaf chloroplasts. Thus, higher plants need to translocate the sulfate from underground to aerial organs, which is accomplished by SULTR transporters [4,53,54]. The sulfate movements between organs, cells, and organelles are strictly regulated by internal and external signals. In the last two decades, the SULTR gene family has been studied much more intensively in model plant *Arabidopsis* and, to varying degrees, in other plant species, such as rice, maize, and cabbage [12,55,56,57]. In this study, a total of 23 *BolSULTR* genes were identified where 2, 4, 15, 15, 9, and 12 SULTRs were found in the genomes of green algae, moss, tomato, potato, maize, and rice, respectively. The results suggested that SULTRs might be encoded by a moderate-sized gene family.

Regardless of the variable size of their genome, the number of *SULTR*s is comparable in the species investigated. Among them, *Zea mays* B84 has the largest genome of 2.1 gigabases (Gb), while only nine *ZmSULTR*s were reported in the maize genome. However, the *Arabidopsis* with the smallest genome of 130 megabases (Mb) contains 12 *SULTR*s. As a close relative of *Arabidopsis*, broccoli belongs to the Brassicaceae family. Due to several rounds of whole genome duplication (WGD) events [47,58,59], the broccoli genome (613 Mb) is nearly five times larger than its *Arabidopsis* counterpart. Interestingly, the members of *BolSULTR* only slightly doubled compared to their *Arabidopsis* counterparts because the phylogenetic analysis did identify duplication events within the *BolSULTR* gene family. It seems that the increased complexity of the genome has little effect on the expansion of the *SULTR* gene family in these plants. Accordingly, the observations might provide a plausible hypothesis for the paradox between the increased genome complexity and the relatively stable copy number of *SULTRs* in diverse species. In this scenario, although several WGD events during Brassicaceae evolution technically increase the copy number of *SULTR*s in the genome, the duplicated *SULTR*s are eventually lost through natural selection that favors the deletion of redundant copies unless the duplicated *SULTR* can acquire new functions [47,60]. Indeed, Ka/Ks ratio analysis indicated that the majority of *BolSULT*Rs have undergone strongly purifying selection in their evolutionary history. However, we are unable to deny other possibilities. For instance, other plant ion transporters, instead of SULTRs, might also have the ability to absorb sulfate from the environment, which might eliminate the need for more SULTRs in these higher plants with more complex genomes. Moreover, Group III represents the largest subfamily of SULTRs according to our analysis, which is in good agreement with previous findings. Although the functional identities of BolSULTR members in Groups II and III have not been determined in broccoli, the increase in *BolSULTR* copies might contribute to the sulfur assimilations and the biosynthesis of glucosinolates and their derivatives because broccoli can accumulate glucosinolates, a group of sulfur-rich secondary metabolites abundantly found in broccoli.

### 4.2. Group II and III SULTRs Might Represent Flowering-Plant-Specific Transporters Associated with Long-Distance Movement of Sulfate

The SULTRs are widely distributed across the kingdom Plantae. In previous studies, two and four SULTRs were reported in *Chlamydomonas reinhardtii* and *moss Physcomitrium patens*, respectively. According to the phylogenetic trees, two green algae SULTRs were present only in Group IV, implying that *SULTR* duplication predated the divergence of unicellular and multicellular life, suggesting that they may represent the ancestral precursors of SULTR found in land plants. Likewise, four bryophyte SULTRs were found in Groups I and IV, suggesting that the SULTR family diverged into at least two groups in the common ancestor of bryophytes. It is noteworthy that all SULTR members in Groups II and III belong to flowering plants, which leads to the suggestion that they might be associated with the long-distance transport of sulfate in plants. In support of this concept, *Arabidopsis SULTR2;1* is specifically expressed in the pericycle and xylem parenchyma cells, controlling the amount of sulfate to be transported from the rhizosphere to the aboveground organs. Similarly, *Arabidopsis SULTR3;5* was found to be preferably expressed in root vasculature, and the *sultr3;5* mutant displayed a severe decrease in root to shoot sulfate movement [19]. Hence, it is not surprising that Group II and II SULTRs are absent in the non-vascular plants moss and algae. Therefore, we hypothesize that the expansion of two groups of SULTRs might have evolved in the vascular lineage as the multicellular land plants need long-distance sulfate transport from root to shoot.

### 4.3. BolSULTR2 Might Be Involved in Plant Defense Against Alternaria brassicicola (Ab), Presumably Through the Regulation of Glutathione Biosynthesis

It has been widely recognized that a sufficient sulfur supply is a prerequisite for plant responses to biotic stresses [29,61]. Because SULTRs are the major contributors to sulfate distributions within plants, they should theoretically impact plant immunity. In our study, the expression levels of several *BolSULTR* genes, including *BolSULTR2;1a/b*, *BolSULTR3;1a/b*, *BolSULTR3;3b*, and *BolSULTR3;4b/c*, were decreased at early stages, although some of them gradually returned to initial levels. Our results support the notion that some *BolSULTR*s might be repressed during the early stage of *Ab* attack, presumably altering the sulfate movements that favor plant immunity. Accordingly, promoter analysis of these *BolSULTR*s revealed the existence of the cis elements related to the MeJA, SA, and ethylene signaling pathways that are implicated in biotic stresses. Consistent with our findings, the expression levels of *OsSultr2;1/2;2* and *OsSultr3;1* were significantly reduced in the rice challenged by the fugal *Magnaporthe grisea* [28]. Similarly, the accumulation of *StuSULTR2* transcripts was reduced significantly when potato leaves were inoculated by the oomycetes *Phytophthora infestans* [62]. Thus, these findings indicate that plants could control sulfate transport through the regulation of SULTR genes, particularly at the early stage of pathogen infections. Similarly, we also observed that transient silencing of *BolSULTR2;1a* resulted in enhanced tolerance to *Ab* in broccoli, implying that BolSULTR2;1a might be a negative regulator of *Ab* resistance. However, stable transgenic lines are still needed to further determine how sulfur metabolism and plant resistance are regulated by *BolSULTR2;1a*. In the future, we will generate the *bolsultr2;1* knockout line through a gene editing method and investigate the distribution of sulfate in *bolsultr2;1* lines.

Glutathione (GSH) is an important sulfur compound and a key cellular redox-buffering tripeptide with irreplaceable roles in plant defense mechanisms. It has been well-established that glutathione is oxidized by reactive oxygen species (ROS) as the antioxidant barrier that ameliorates oxidative stress imposed on sensitive cellular components [63,64,65]. GSH acts as a non-enzymatic antioxidant that directly removes reactive oxygen species (ROS) and works in concert with enzymatic antioxidant systems, such as the ASA–GSH cycle. For example, ascorbate peroxidase (APX) relies on GSH to regenerate ascorbic acid (ASA), and elevated levels of GSH may enhance APX activity, facilitating the co-removal of hydrogen peroxide (H_2_O_2_) [66]. In our study, the GSH content of the *BolSULTR2;1*-silencing in the leaves was much higher in comparison to the wild-type and TRV:00 lines. As expected, elevated levels of GSH can enhance antioxidant enzymes’ activity to work together to remove ROS, thereby protecting cells from oxidative stress, which provides an explanation for the *BolSULTR2;1*-silencing broccoli’s increased resistance to *Ab*.

The *SULTR2.1* gene is involved in long-distance sulfate transport between leaves in *Arabidopsis*, and it is likely that *BolSULTR2;1s* might have similar capabilities in controlling sulfate movement. Therefore, our results perhaps show that upon *Ab* attack, broccoli leaves reduce the expression of *BolSULTR2;1s*, which transitorily reduces sulfate movement to non-infected counterparts. It is thus reasonable to assume that the accumulation of intracellular sulfate is conducive to elevated GSH biosynthesis during early infection, thus conferring increased tolerance to oxidative injuries imposed by *Ab* attack.

## 5. Conclusions

Sulfur is an essential element in plant growth resistance, and plant sulfur transporters play an essential role in the acquisition and distribution of sulfur. In this study, we identified 23 *BolSULTR* genes from the *Brassica oleracea* genome, classified and named them, studied the evolutional relationship, gene structure, and cis-regulatory elements of *BolSULTRs* in detail, and predicted the interaction proteins of the *BolSULTR* families. We characterized the expression patterns of *BolSULTRs* in different tissues and under different biological stresses. The VIGS experiment confirmed that silencing of *BolSULTR2;1* resulted in enhanced tolerance to *Ab* by regulating GSH biosynthesis. These findings provide new insights into broccoli sulfate transporters in plant disease tolerance. Furthermore, functional studies are needed to reveal the molecular mechanism of *BolSULTRs* in sulfate transport and *Ab* infections.

## Figures and Tables

**Table 1:** Physicochemical properties of SULTR protein in broccoli.

Gene_Name ^a^	Gene_ID ^b^	Putative Proteins’ Physical and Chemical Parameters ^c^					Predicted Location(s) ^d^
		Peptide Length (aa)	MW (kDa)	pI	Instability Index	GRAVY	
*BolSULTR1.2b*	BolC02g034190.2J	652	71.64	9.12	33.97	0.410	Cell membrane
*BolSULTR1;1*	BolC02g019620.2J	648	70.59	9.12	35.14	0.406	Cell membrane
*BolSULTR1;2a*	BolC06g049010.2J	655	71.84	8.81	34.81	0.401	Cell membrane
*BolSULTR1;3*	BolC05g020480.2J	683	74.96	9.22	34.71	0.456	Cell membrane
*BolSULTR2;1a*	BolC02g004100.2J	677	73.97	9.04	37.58	0.427	Cell membrane
*BolSULTR2;1b*	BolC09g062520.2J	686	75.39	8.86	39.45	0.414	Cell membrane
*BolSULTR2;1c*	BolC09g062540.2J	629	68.97	8.21	37.44	0.473	Cell membrane
*BolSULTR2;1d*	BolC09g062560.2J	624	68.22	8.54	36.2	0.489	Cell membrane
*BolSULTR2;2*	BolC06g049000.2J	471	51.29	9.43	30.44	0.586	Cell membrane
*BolSULTR3;1a*	BolC07g044940.2J	759	83.77	8.99	33.55	0.263	Cell membrane
*BolSULTR3;1b*	BolC08g036100.2J	876	97.30	5.96	40.41	−0.093	Cell membrane, nucleus
*BolSULTR3;1c*	BolC04g040390.2J	390	43.33	8.5	40.64	0.407	Cell membrane
*BolSULTR3;2*	BolC09g002080.2J	650	71.74	8.81	42.51	0.438	Cell membrane
*BolSULTR3;3a*	BolC07g020360.2J	656	72.22	9.1	35.68	0.461	Cell membrane
*BolSULTR3;3b*	BolC05g021440.2J	632	69.42	9.3	36.37	0.425	Cell membrane
*BolSULTR3;4a*	BolC05g050200.2J	656	71.95	9.51	40.83	0.407	Cell membrane
*BolSULTR3;4b*	BolC01g046840.2J	660	72.09	9.63	39.27	0.409	Cell membrane
*BolSULTR3;4c*	BolC03g044170.2J	653	71.60	9.54	37.55	0.44	Cell membrane
*BolSULTR3;5a*	BolC03g010280.2J	636	70.53	8.48	35.85	0.442	Cell membrane
*BolSULTR3;5b*	BolC02g009920.2J	634	70.38	8.85	32.21	0.447	Cell membrane
*BolSULTR4;1a*	BolC03g006540.2J	692	75.88	8.64	45.62	0.324	Chloroplast
*BolSULTR4;1b*	BolC09g059530.2J	693	75.89	8.34	43.5	0.288	Chloroplast
*BolSULTR4;2*	BolC03g041900.2J	989	108.00	6.03	39.73	0.138	Chloroplast, vacuole

^a^, Name refers to systematic designation of members of the *BolSULTR* family according to the homology against *Arabidopsis*. ^b^, Gene accession number in BRAD database. ^c^, The various physical and chemical parameters calculated from the protein sequences we entered. ^d^, Subcellular localization was predicted using Plant-mPLoc.

## Data Availability

The original contributions presented in this study are included in the article/Supplementary Materials; further inquiries can be directed to the corresponding author.

## Supplementary Materials

The following supporting information can be downloaded at https://www.mdpi.com/article/10.3390/antiox14040496/s1, Figure S1: The 23 BolSULTR members were named according to their homology with their *Arabidopsis* counterparts; Figure S2: The conserved regions of Sulfate_transp (PF00916) or STAS (PF01740) were visualized on the ChiPlot; Figure S3: Distribution map of promoter action elements for 2 kb upstream *BolSULTR* genes; Figure S4: Highly homologous gene sequences of *BolSULTR2;1a*; Figure S5: Schematic diagram of contagious disease; Table S1: The 23 BolSULTR members were identified and named according to their homology with their Arabidopsis counterparts; Table S2: The BolSULTR gene pairs had a Ka/Ks ratio and duplication type; Table S3: The relevant primer sequences; Table S4: RNA-seq datasets of Brassica from the GSE42891 (GEO database); Table S5: RNA-seq data of *BolSULTR* genes from leaves at five time points under Alternaria brassicicola stress in broccoli susceptible cultivar ‘41-3’; Table S6: Browse interactions in tabular form (lists only one-way edges: A-B).
