# Supplementary material for: Genome-Wide Identification of the Sulfate Transporter Gene Family Reveals That BolSULTR2;1 Regulates Plant Resistance to Alternaria brassicicola Through the Modulation of Glutathione Biosynthesis in Broccoli
Source: Antioxidants (Basel). 2025 Apr 20;14(4):496. doi: 10.3390/antiox14040496 (PMC12024372; doi:10.3390/antiox14040496)
Supplement: Supplementary file 1 [file antioxidants-14-00496-s001.zip › Figures S1-S5.pdf]

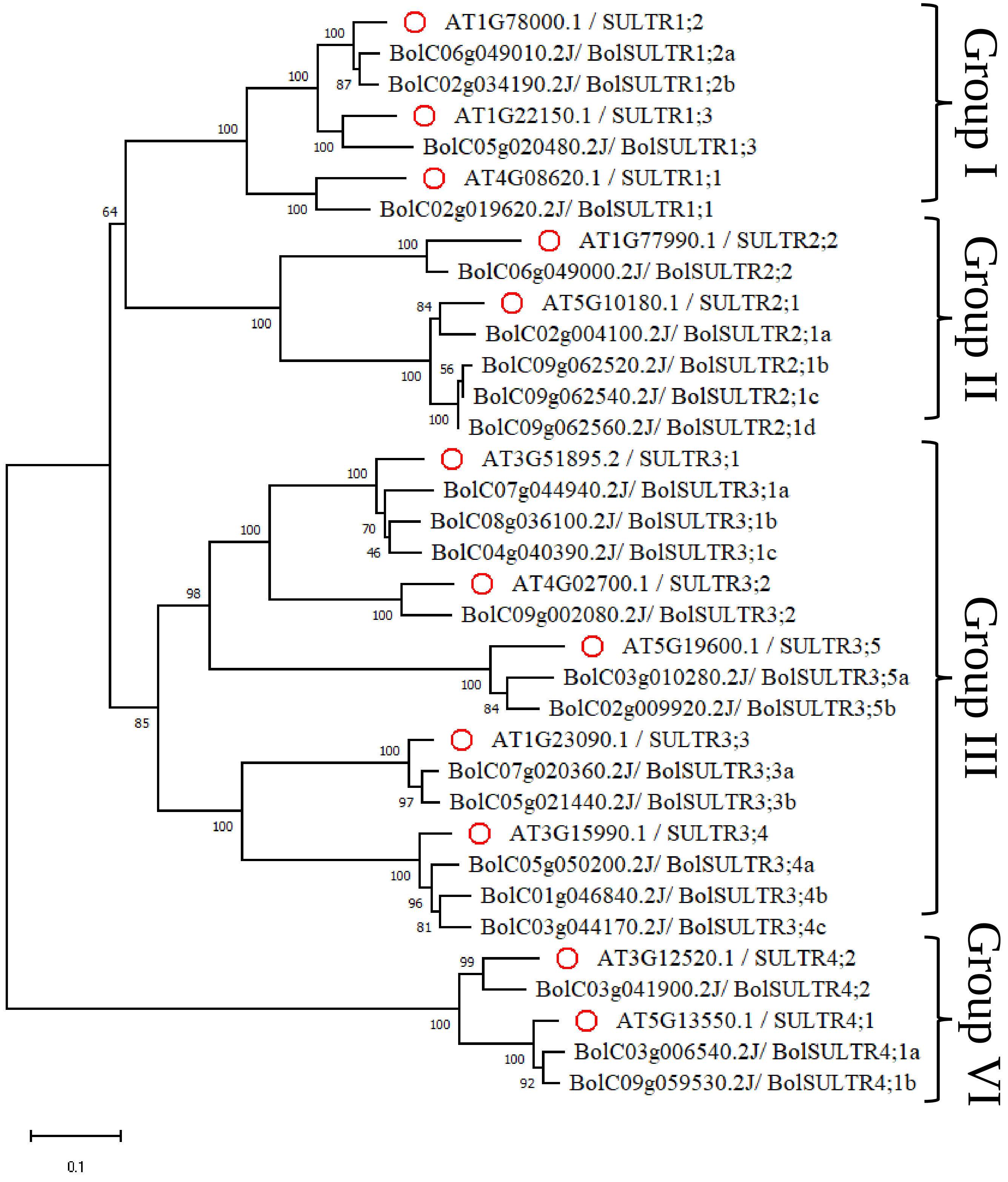

Figure S1. The 23 *BolSULTR* members were named according to the homology with *Arabidopsis* counterparts. Red circles represent *Arabidopsis* sequences of SULTR used as seed sequences.

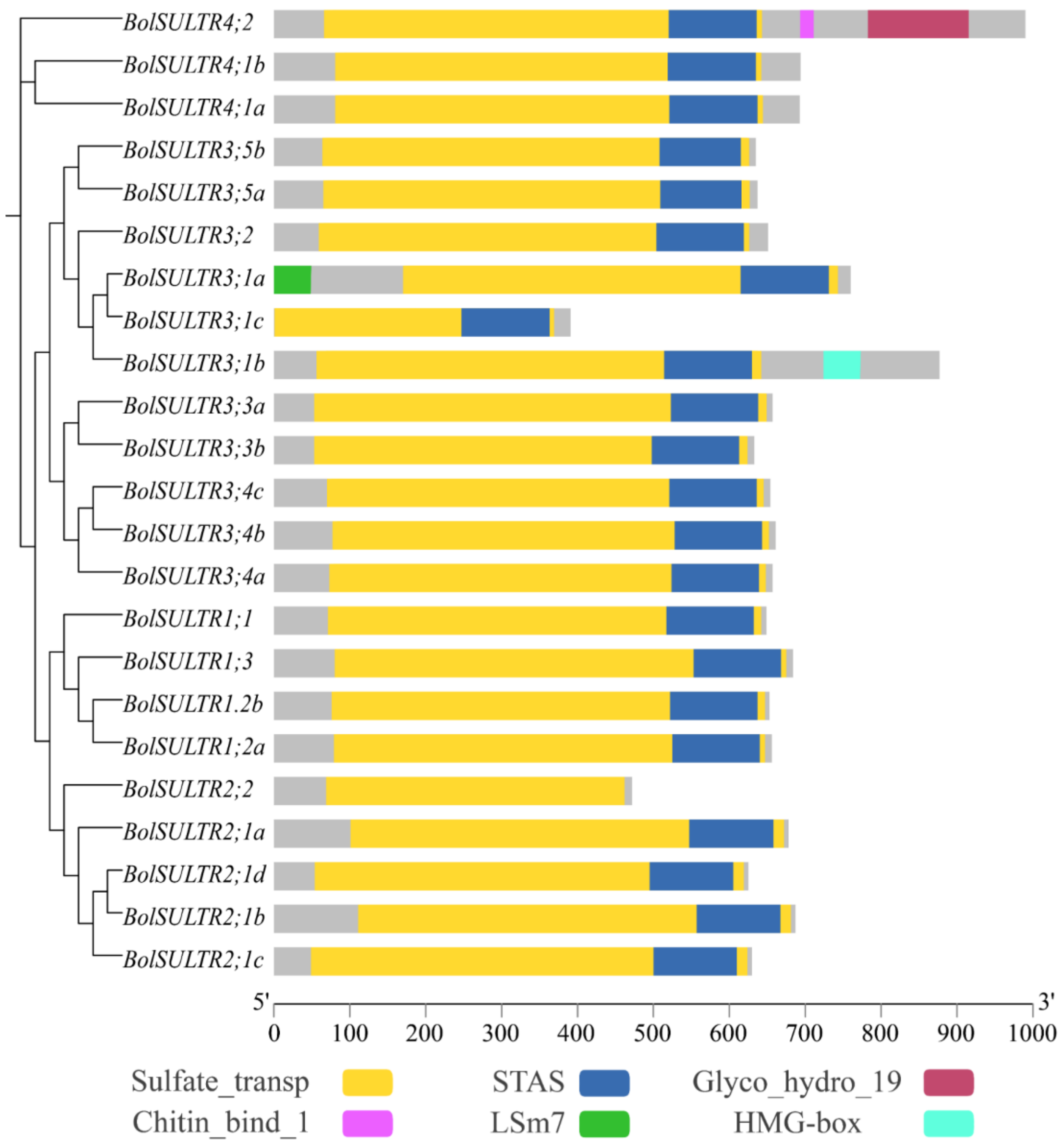

Figure S2. The conserved regions of Sulfate_transp (PF00916) or STAS (PF01740) were visualization on the ChiPlot. (A) Creating a phylogenetic tree involves arranging 23 BolSULTR conserved regions based on their evolutionary relationships (B) Schematic organization of conserved domains in BolSULTR proteins.

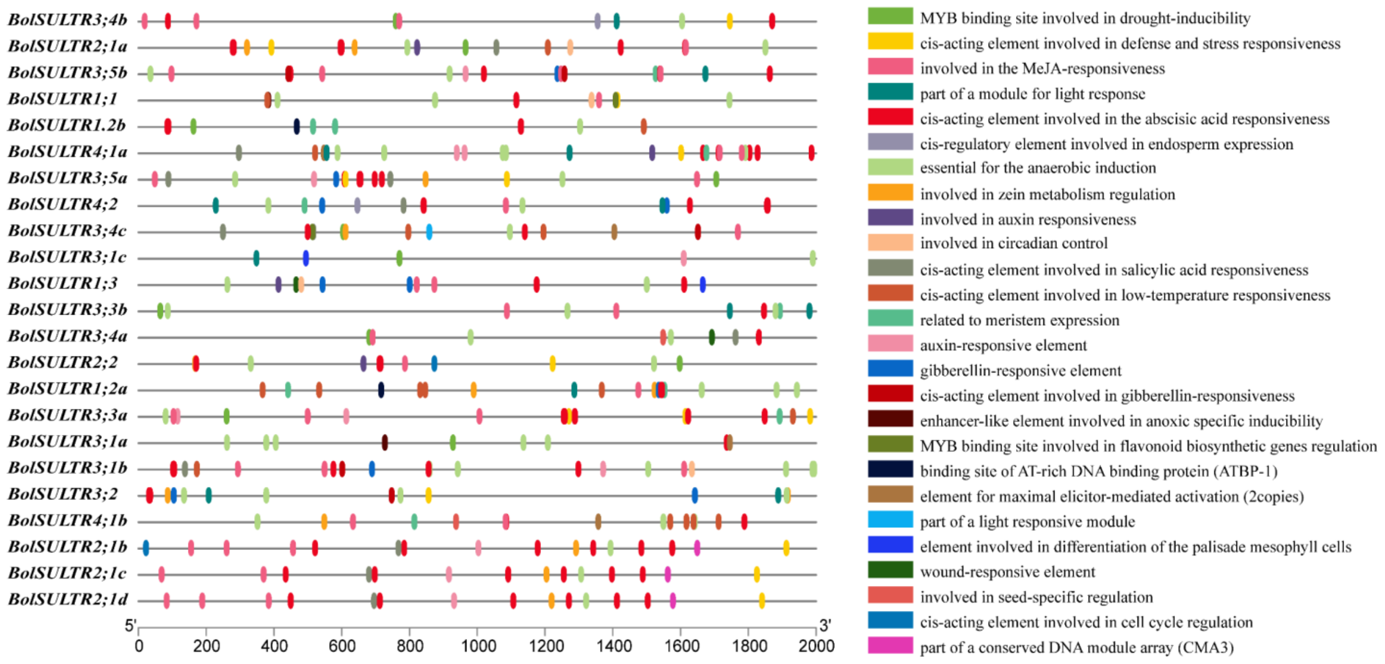

Figure S3. Distribution map of promoter action elements for 2 kb upstream *BolSULTR* genes.

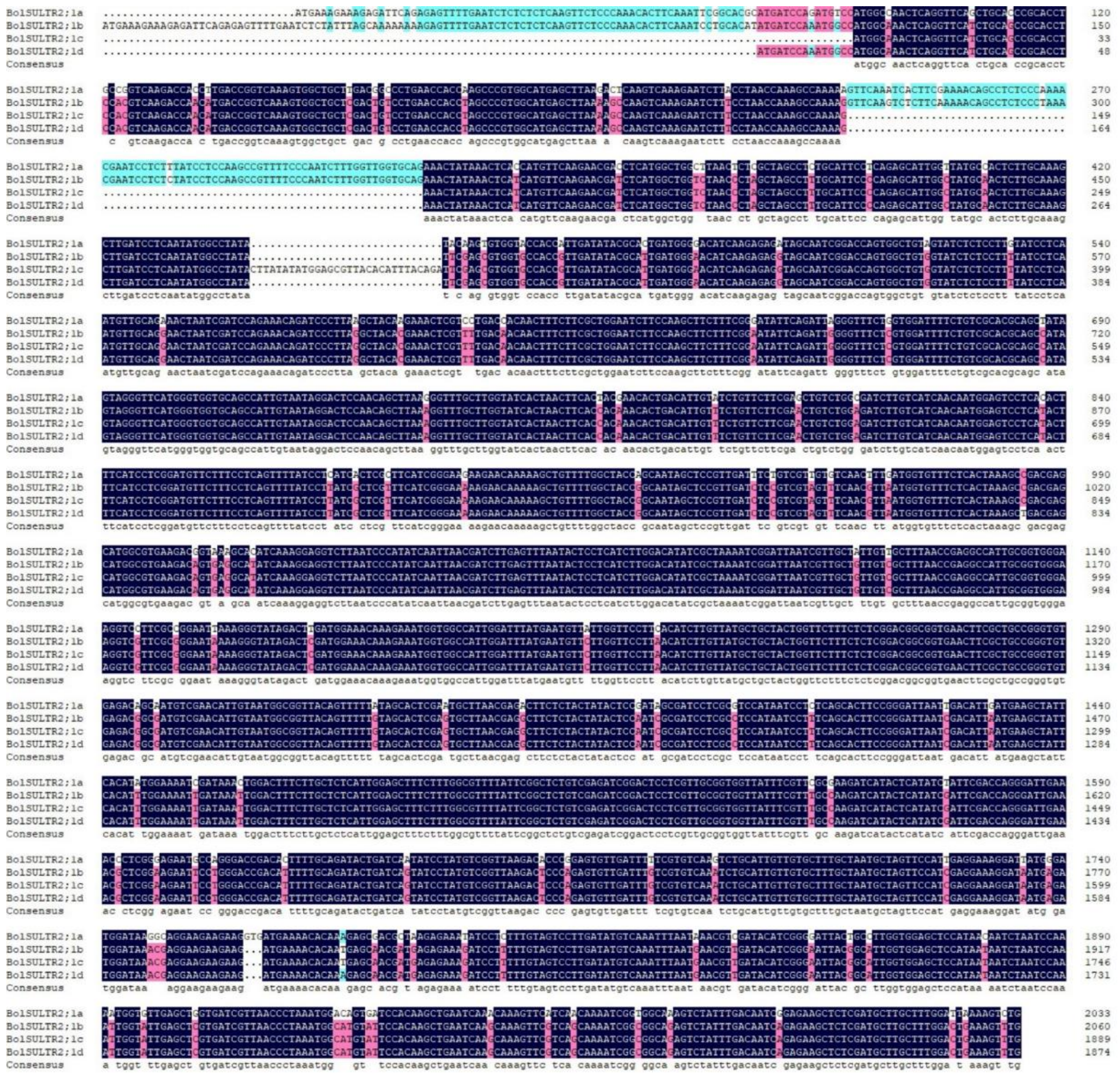

Figure S4. Highly homologous gene sequences of *BolSULTR2;1a*.

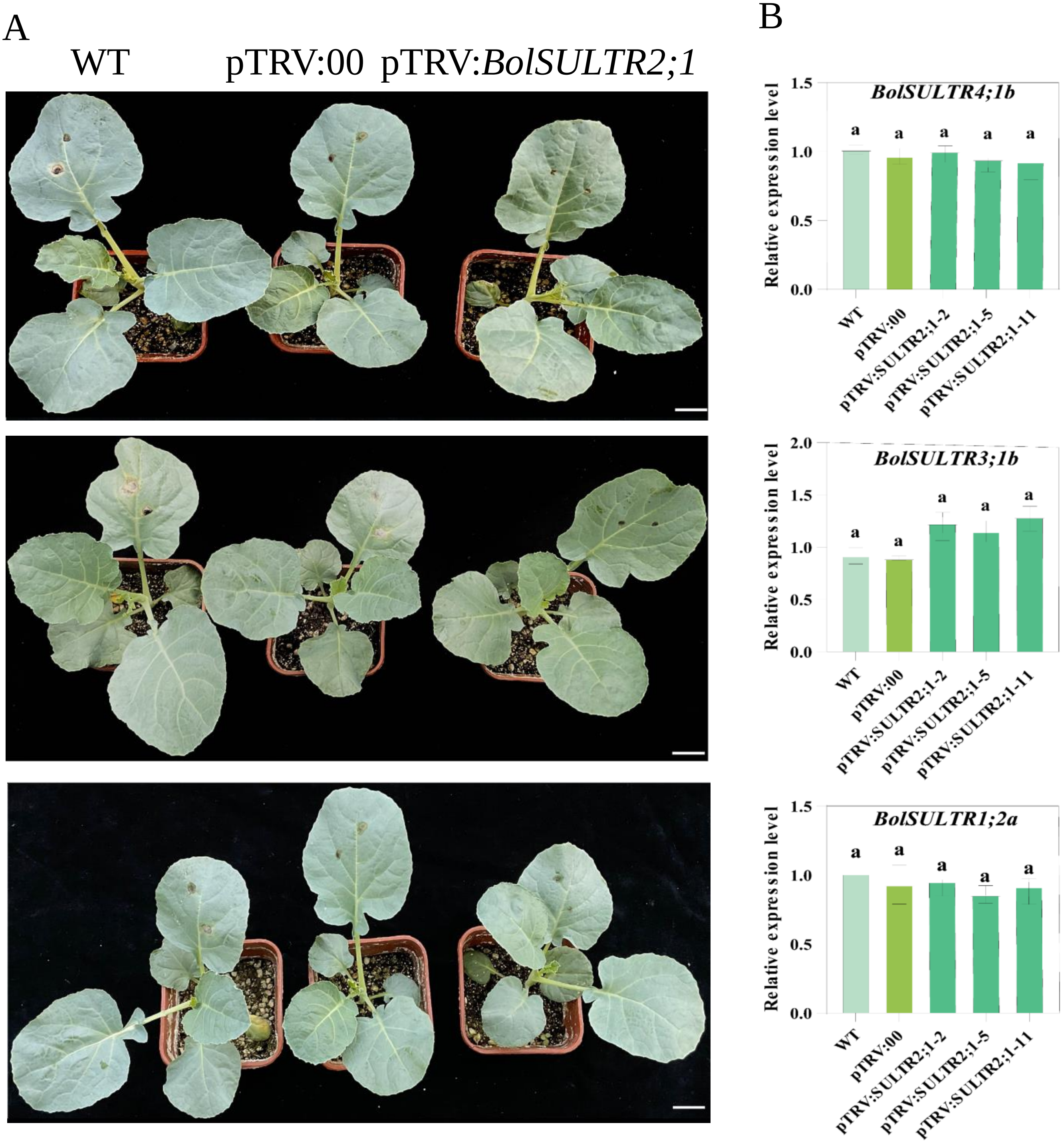

Figure S5

Figure S5. Schematic diagram contagious disease. (A) Disease injury in *BolSULTR2;1*-silencing and control plants after five days inoculate *Ab* treatments. Bar = 2 cm.(B) There was no difference in expression of expression levels of other *BolSULTR* genes in silent and control plants. Error bars indicate SE from three biological replicates, and different letters indicate significant differences ($p < 0.05$).
